# Delayed Corneal Epithelial Healing after Intravitreal Bevacizumab: A Clinical and Experimental Study

**Published:** 2011-01

**Authors:** Gustavo A Colombres, Ana L Gramajo, Maria P Arrambide, Silvina M Juarez, J Fernando Arevalo, Jorge Bar, Claudio P Juarez, Jose D Luna

**Affiliations:** 1 Centro Privado de Ojos Romagosa-Fundación VER, Córdoba, Argentina; 2 Clinica Oftalmologica Centro Caracas, Caracas, Venezuela; 3 Departamento de Oftalmología, Hospital Nacional de Clínicas, Buenos Aires, Argentina

**Keywords:** Intravitreal Bevacizumab, Corneal Epithelial Defect, Corneal Edema, Antiangiogenics, Complications

## Abstract

**Purpose:**

To report corneal epithelial defects (CEDs) and delayed epithelial healing after intravitreal bevacizumab (IVB) injection and to describe delayed corneal epithelial healing with topical administration of bevacizumab in an experimental rabbit model.

**Methods:**

A retrospective chart review was performed on 850 eyes of 850 patients with neovascular eye disease and diabetic macular edema who had received 1.25 to 2.5 mg IVB. In the experimental arm of the study, photorefractive keratectomy was used to create a 3 mm CED in the right eyes of 18 New Zealand rabbits which were then randomized to three equal groups. All rabbits received topical antibiotics, additionally those in group A received topical bevacizumab and animals in group B were treated with topical corticosteroids. The rate of epithelial healing was assessed at different time points using slitlamp photography.

**Results:**

In the clinical study, seven eyes of seven subjects developed CEDs the day after IVB injection. All of these eyes had preexisting corneal edema. The healing period ranged from 3 to 38 days (average 11 days) despite appropriate medical management. In the experimental study, topical bevacizumab and corticosteroids both significantly hindered corneal epithelial healing at 12 and 24 hours.

**Conclusion:**

Bevacizumab was demonstrated to cause CEDs in clinical settings. Moreover, corneal epithelial healing was delayed by topical application of bevacizumab, in the experimental model. These short-term results suggest that corneal edema may be considered as a risk factor for epithelial defects after IVB.

## INTRODUCTION

The vascular endothelial growth factor (VEGF) has been considered as the major angiogenic stimulus for ocular neovascularization.^1^–^6^ Bevacizumab (Avastin), a full - length humanized monoclonal antibody, binds to and inhibits all biologically active isoforms of VEGF-A.^1^,^7^ Although FDA-approved only for intravenous administration in the treatment of metastatic colorectal cancer and other malignant tumors, this drug is widely used off-label in various ophthalmic conditions. An intraocular injection of 1.25 mg/0.05 ml bevacizumab has been reported to markedly promote regression of ocular neovascularization in experimental and clinical studies.^8^,^9^ Several reports have demonstrated that this drug can be injected safely and effectively into the eye for treatment of a variety of ocular disorders such as age-related macular degeneration (AMD), proliferative diabetic retinopathy (PDR) and neovascular glaucoma (NVG), among others.^10^–^12^ However in 2006, adverse drug-related events associated with intravitreal bevacizumab (IVB) treatment were reported in retrospective studies on a limited number of patients.^13^,^14^

Several growth factors have been implicated in corneal epithelial repair.^15^ Cytokines, growth factors, and matrix-degrading enzymes have an important role in ocular surface inflammation, wound healing, and angiogenesis. Recently, several reports have suggested that small doses of bevacizumab, delivered topically or subconjunctivally for treatment of corneal neovascularization and pterygia, do not cause serious adverse effects.^16^–^19^ On the other hand, it has been shown that the human corneal epithelium expresses VEGF-A and its receptors.^20^ These observations may suggest a role for VEGF-A not only in corneal neovascularization, but also in other physiologic or pathologic conditions such as corneal wound healing.

The present study was designed to determine the effect of IVB on the corneal epithelium and to assess, in an experimental model, the time required for corneal epithelial healing after exposure to topical bevacizumab.

## METHODS

### Clinical Study

A retrospective chart review was performed on 850 eyes of 850 individuals including 548 male and 302 female subjects with mean age of 69±19 (range 29 to 73) years who had received IVB from January 1, 2006 to January 1, 2009 for different ocular conditions including AMD (546 eyes), diabetic macular edema (DME, 259 eyes), and NVG secondary to PDR (32 eyes) or central retinal vein occlusion (13 eyes). All patients who received IVB during this time period were included in the review, without exceptions. The patients had received 1.25 to 2.5 mg of intraocular bevacizumab.

The study received Institutional Review Board approval from the Oulton-Romagosa Joint Committee on Clinical Investigation (CIEIS Oulton-Romagosa) and each patient signed a written informed consent form prior to treatment.

### Experimental Study

Eighteen male New Zealand rabbits (average weight 345±42 g) were kept under a 12 hour light/12 hour dark schedule with free access to food and water. All experiments were conducted in accordance with the Association for Research in Vision and Ophthalmology (ARVO) Statements for the Use of Animals in Ophthalmic and Vision Research.

The rabbits were anesthetized using intramuscular xylazine hydrochloride (5 mg/ kg of body weight) and ketamine hydrochloride (35 mg/kg of body weight). After topical application of 1% tetracaine chlorhydrate (Anestalcon, Alcon Laboratories, Fort Worth, TX, USA) and 5% povidone iodine (Betadine, Alcon Laboratories, Fort Worth, TX, USA), photorefractive keratectomy (PRK) was performed to induce a central corneal lesion, 3 mm in diameter and 120 μm in depth, in the right eye of all 18 animals using the Compak-200 MiniExcimer laser (LaserSight Technologies Inc., Winter Park, FL, USA).

The rabbits were randomly allocated into 3 groups of 6 animals. All three groups were treated with topical 0.3% ofloxacin eye drops four times daily. Additionally, groups A and B received 2.5 mg/ml topical bevacizumab (Avastin, Genetech Inc., San Francisco, CA, USA) and 1% prednisolone acetate (Prednefrin Forte, Allergan, Irvine, CA, USA) eye drops twice a day, respectively. Within each study group, the fellow untreated eye received the same medical regimen employed for the right eye. Bevacizumab eye drops were prepared from the commercially available solution diluted in 0.9% preservative-free artificial tears to reach a concentration of 2.5 mg/ml.

Measurement of corneal epithelial defects (CEDs) was performed after fluorescein staining using slitlamp photography (SL-7E, Topcon, Tokyo, Japan) at different time points after injury (12, 24, 48 and 72 hours), until the lesions were fully healed. Images were evaluated using an analysis software (Image Analyzer 1.32). All observations were performed by a single masked examiner.

Data were analyzed statistically using GraphPad Prism version 3.0 (GraphPad Software Inc., San Diego, CA, USA). The Newman-Keuls multiple comparison test was used to compare data within each experiment and the unpaired t-test was used to compare data between two variables. P-values less than 0.05 were considered as statistically significant.

## RESULTS

### Clinical Study

CEDs developed in 7 patients treated with IVB. Six of them were diabetic (85.7%), of whom 4 subjects had NVG, 4 had PDR and 1 had DME. The single remaining patient had been treated for AMD (Table 1). All patients had preexisting corneal edema, including the AMD patient who had concomitant Fuchs’ corneal endothelial dystrophy.

The dose of IVB ranged from 1.25 mg to 2.5 mg (Table 1). All 7 eyes developed CED the day after uneventful IVB injection. None of the patients without corneal edema developed such a complication and it is worth noting that although 45 patients with NVG were treated, only eyes with corneal edema developed CEDs regardless of the dose and volume of IVB, or intraocular pressure (IOP) level. After the development of CED, all cases were treated with occlusion (eye patching or therapeutic contact lenses) and topical antibiotics. The healing period ranged from 3 to 38 days (mean, 11 days). The condition was further complicated in one eye by an infectious keratitis, 3 days after IVB, which was successfully treated with topical vancomycin.

### Experimental Study

**Figure 1:**
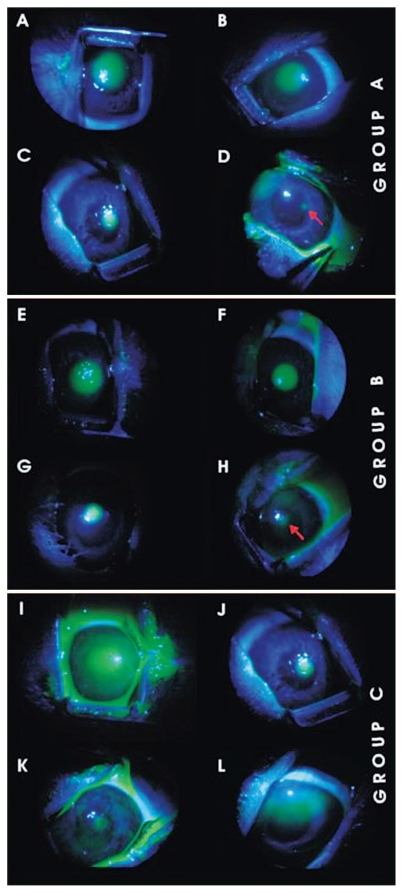
Clinical stages of CED healing times in rabbit corneas after exposure to different medications following PRK. Figures 1A, B, C, and D show corneas treated with topical bevacizumab in addition to antibiotic eye drops (ofloxacin) immediately after PRK, at 12 hours, 24 hours, and 48 hours following treatment (group A). Figures 1E, F, G, and H correspond to group B, which received topical steroids in addition to topical ofloxacin following PRK. Figure 1I, J, K, and L (group C) received only topical ofloxacin. These figures correspond to CEDs immediately after PRK treatment, at 12 hours, 24 hours, and 48 hours following PRK.

**Figure 2:**
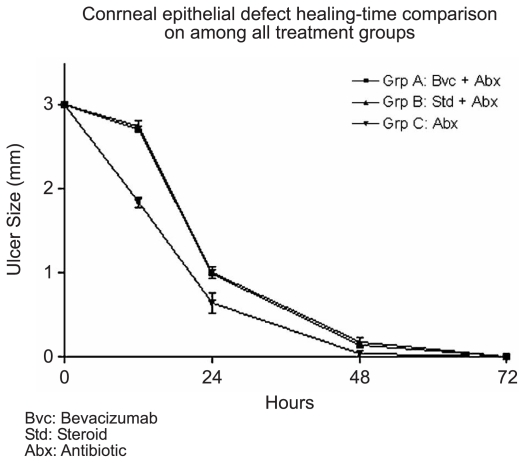
Comparison of CED healing time among all treatment groups.

**Figure 3:**
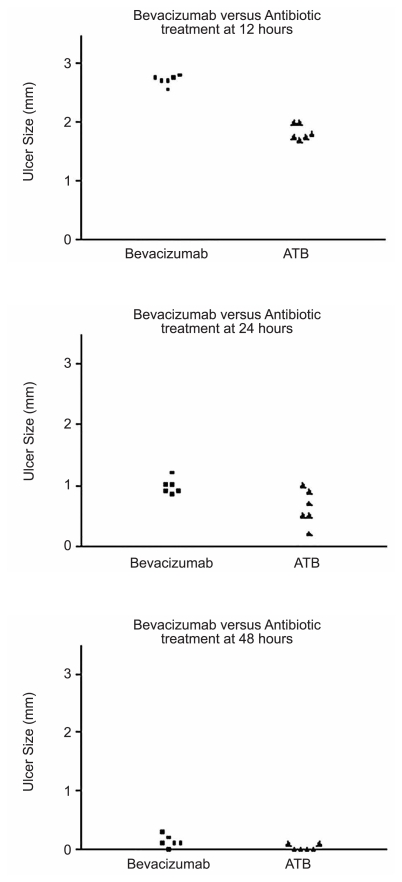
Comparison of CED healing time between the bevacizumab and antibiotics groups at different time points.

**Figure 4:**
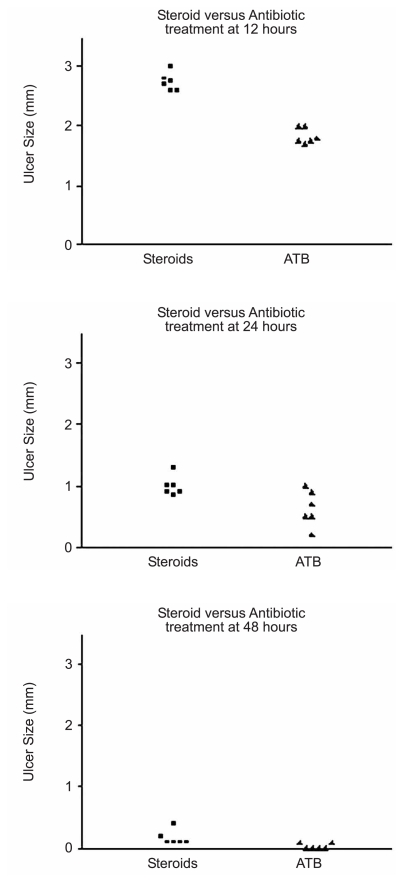
Comparison of CED healing time between the steroid and antibiotics groups at different time points.

**Figure 5:**
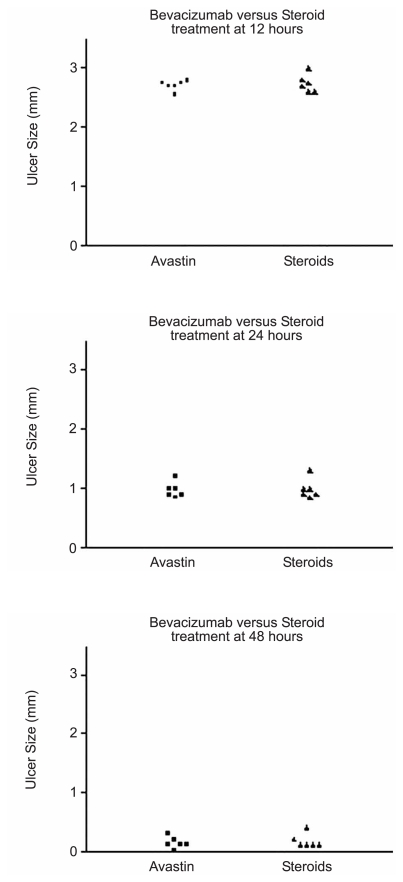
Comparison of CED healing time between the bevacizumab and corticosteroid groups at different time points.

Figures 1 through 4 depict a delay in wound healing in rabbit eyes treated with bevacizumab and corticosteroids (groups A and B, respectively) as compared to those treated with antibiotics alone (group C). Complete healing of the cornea was observed after 48 hours in group C (Figures 1 and 2). However, bevacizumab and corticosteroid-treated corneas healed after 72 hours. Twelve hours after PRK, a significant difference in healing was observed between groups A and B as compared to group C (both P values < 0.0001); groups A and B, however were comparable (P=0.65). Similar intergroup differences were observed at 24 hours (groups A and C, P=0.02; groups B and C, P=0.02; groups A and B, P=0.85; Figures 3, 4, and 5).

Corneas without PRK (the untreated fellow eyes) were not affected by any of the three treatment regimens.

## DISCUSSION

Increased vascular permeability and neovascularization are major causes of visual loss in exudative AMD and diabetic retinopathy. Although anti-VEFG therapy has yielded promising results for treatment of these diseases,^12^,^21^ complications have been reported.^22^ Wong et al^22^ reported three eyes with adverse corneal events after IVB. In one of these cases, corneal edema developed 30 days after the injection.

In the current study seven eyes developed CEDs 24 hours after injection of different doses of IVB. All eyes demonstrated delayed healing even though intensive occlusive treatment was performed in all cases. A common factor in all patients was preexisting corneal edema. Although other risk factors for development of corneal ulceration (such as the use of antiseptic solutions or elevated IOP due to IVB injection) existed in this setting, it is worth noting that none of the eyes without corneal edema developed CEDs. Regarding IOP elevation, it is interesting that although 45 patients with NVG were treated, only eyes with corneal edema developed corneal ulceration. This occurred regardless of the dose and volume of IVB or IOP level.

Corneal stromal and epithelial edema, lead to attenuation or rupture in Bowman’s layer and the epithelial basement membrane.^23^,^24^ This predisposes to poor epithelial adhesion and formation of subepithelial bullae which in turn cause persistent epithelial defects. A vicious cycle of epithelial breakdown and wound healing produces a state of chronic inflammation. Inhibition of VEGF may adversely affect corneal physiology and predispose to development of an inflammatory state and impaired wound healing.

There is a possibility of mild reflux of bevacizumab from the intraocular compartment onto the ocular surface after IVB is injected. This scenario is more likely to occur in eyes with high IOP, since intraocular pressure has been described, among several other factors, to influence the development of reflux.^25^ It is worth noting that 4 of the 7 patients who developed CED presented with NVG and high IOP levels.

We demonstrated experimentally that topical bevacizumab delayed wound healing in rabbit corneas in a PRK model, which was comparable to the effect of topical corticosteroids.

Following corneal injury, a combination of rapid signal transduction events and cell migration are essential for wound healing.^26^ This involves epithelial proliferation, migration and stratification, and stromal wound healing.^27^ Cytokines, growth factors, and several matrix degrading enzymes participate in tissue degradation, which is associated with corneal inflammation and wound healing.

VEGF and the vascular endothelial growth factor receptor 2 (VEGFR-2) are active in the epithelial wound healing phase, and are expressed in proliferating corneal epithelium, keratocytes, and endothelium.^28^ Gan et al^28^ showed that expression of VEGF decreased and nearly disappeared in the corneal epithelium immediately peripheral to the leading edge, while at the wound edge epithelial cells strongly expressed this receptor. There is a possibility that topical bevacizumab applied on the wounded rabbit corneas inhibited the effect of VEGF on the proliferating epithelium, thus delaying healing. In clinical settings, the intravitreal injection of bevacizumab may have caused spillage over the ocular surface and created the same effect on the edematous corneas of our seven patients.

Another possible explanation for the delay in healing could be related to the effect of bevacizumab on corneal nerves during wounding. Corneal nerves are of great importance due to their role in protecting the cornea from irritants and because of their trophic properties, which are necessary to maintain a healthy ocular surface.^29^ Disruption of corneal nerves has been shown to impair corneal healing significantly.^30^ VEGF has also been demonstrated to be an important factor for nerve growth; *in vitro* experiments have shown that VEGF and its receptor are expressed by neurons, and that they stimulate neurogenic, neuroprotective, and neurotrophic activities.^31^ VEGF may also have an important role in corneal healing, possibly by promoting or guiding nerve growth. Inhibition of VEGF has previously been shown to reduce the serum dependent growth of cultured neurons by 17%. More importantly, by adding VEGF neutralizing antibodies to corneas undergoing sub-basal nerve plexus regeneration, neuronal repair was inhibited by 23%.^32^

Corneal wound healing is associated with expression of adhesion molecules, such as integrins that mediate interactions between contiguous cells and/or the extracellular matrix (ECM) and thus, modify cellular properties such as proliferation, migration, differentiation and apoptosis.^33^ A recent report demonstrated that bevacizumab delayed corneal wound healing and inhibited the expression of integrin.^34^ Integrin alpha 5 beta 1 is the classical receptor for fibronectin and under optimal conditions, mediates cellular attachment to the ECM.^35^ VEGFR-1, a soluble tyrosine kinase receptor for VEGF, is present within the extracellular matrix and is able to interact with alpha 5 beta 1 integrin. Moreover, alpha 5 integrin has been reported to enhance migration by binding directly to VEGFR-1.^36^ Inhibition of VEGF probably interferes with the normal function of fibronectin and disturbs cellular attachment of the corneal epithelium to Bowman layer and thus, delays corneal re-epithelialization.

To our knowledge, the current study is the first to report corneal edema as a risk factor for development of corneal epithelial defects after IVB injections. Delayed epithelial healing was probably related to bevacizumab as demonstrated experimentally in our rabbit model of PRK-induced CED. Although certain pathways involved in corneal epithelial healing, cell adhesion and neurotrophic mechanisms may play a role, the precise mechanisms of this inhibition are not yet well established. Physicians should bear in mind that this drug can delay wound healing and should therefore be cautious when applying bevacizumab to any compromised ocular surface.

## Figures and Tables

**Table 1:** Clinical data of seven subjects who developed epithelial defects after IVB

Patient	Age/Sex	Previous Diagnosis	IVB Dose (mg/ml)	Healing Time (days)	Complications
1	72/F	AMD	1.25	7	None
2	56/F	Diabetes (NVI)	1.25	10	None
3	62/M	CRVO (NVI)	2.5	7	None
4	52/M	Diabetes (NVI)	2.5	5	None
5	62/F	Diabetes (NVI)	2.5	7	None
6	64/F	Diabetes (DME)	2.0	3	None
7	57/F	Diabetes (PDR)	2.0	38	Infectious keratitis

IVB, intravitreal bevacizumab; F, female; M, male; AMD, age-related macular degeneration; NVI, iris neovascularization; CRVO, central retinal vein occlusion; DME, diabetic macular edema; PDR, proliferative diabetic retinopathy
